# Innovative competency-based education approach to radiology residency: integrating modular training in clinical practice and research

**DOI:** 10.1186/s13244-026-02335-z

**Published:** 2026-06-25

**Authors:** Shang Wan, Lei Ye, Xibiao Yang, Zhengyan Li, Na Hu, Bin Song, Ningna Fan, Xijiao Liu, Zixing Huang

**Affiliations:** 1 Department of Radiology, West China Hospital, Sichuan University, Chengdu, China; 2 Continuing Medical Education Department, West China Hospital, Sichuan University, Chengdu, China

**Keywords:** Radiology, Residency training, Competency-based education, Curriculum reform, Disease-oriented modular curriculum

## Abstract

**Objectives:**

This study aimed to design and evaluate a competency-based education (CBE)-oriented modular training model for radiology residents, emphasizing interdisciplinary integration and disease-focused instruction to enhance clinical competence.

**Materials and methods:**

This prospective controlled study included 75 radiology residents enrolled in standardized training from September 2020 to July 2024. A CBE-oriented modular training model was developed, structured around approximately 27 radiologic subspecialties and incorporating interdisciplinary instruction, clinical team rotations, and image post-processing skill training. The participants were assigned to either the experimental group (CBE training) or the control group (traditional training). Teaching effectiveness was evaluated by comparing theoretical and practical exam scores via statistical analysis.

**Results:**

Overall, there were no significant differences in theoretical (83.26 ± 6.99 vs 81.76 ± 7.54; *p* > 0.05) or practical scores (86.79 ± 8.90 vs 84.91 ± 10.71; *p* > 0.05) between the two groups. However, subgroup analysis revealed that, among residents in the non-graduate subgroup, the experimental group achieved significantly higher theoretical (*p* = 0.0179) and practical scores (*p* = 0.028) than the control group. No significant differences were observed in the enrolled-graduate subgroup (*p* > 0.05).

**Conclusions:**

The competency-based modular training model significantly improved clinical competence among residents in the non-graduate subgroup but showed less additional benefit in the enrolled-graduate subgroup. This study offers insights for advancing radiology residency training reform.

**Critical relevance statement:**

The distinct effectiveness of this approach was particularly beneficial for trainees with diverse educational backgrounds. This training model enhances residents’ clinical competence and research capabilities and provides insights for global radiology education reform, aligning training paradigms with evolving clinical and technological advancements.

**Key Points:**

- A CBE-oriented modular training model for radiology residents was developed, integrating interdisciplinary learning, team-based clinical training, and image post-processing instruction.
- Although no overall difference in examination scores was observed between the two groups, residents in the non-graduate subgroup achieved significantly better theoretical and practical scores under the CBE model.

**Graphical Abstract:**

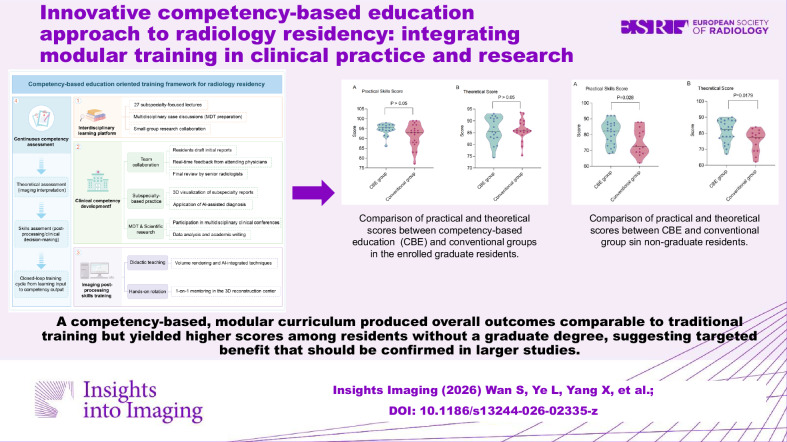

## Introduction

Radiology is a vital component of modern medicine, playing a critical role in the diagnosis, treatment, and prognosis assessment of disease. With the rapid advancement of medical imaging technologies, particularly the widespread application of artificial intelligence (AI) and three-dimensional image processing, the scope and depth of radiology continue to expand, and the demands on radiology professionals have become increased accordingly. Traditionally, radiology residency training in China has focused primarily on didactic instruction, typically comprising systematic lecture-based courses in radiologic theory, clinical rotations, and basic training in imaging techniques [1, 2]. However, this conventional model often neglects the systematic development of residents’ hands-on procedural skills, clinical decision-making skills, research capacity, and interdisciplinary communication skills [3]. Consequently, when faced with complex clinical problems, many radiology residents struggle to make rapid and accurate diagnostic decisions and have difficulty keeping up with the latest advances in imaging, thereby limiting improvements in the quality of patient care [3].

In recent years, there have been substantial efforts worldwide to reform radiology residency training. Leading medical education programs in other countries have begun implementing competency-based education (CBE) models in radiology. These models design curricular content and assessments around the competencies and standards that trainees must achieve in future practice, emphasizing the development of residents’ clinical performance, research capability, and lifelong learning habits. CBE-based approaches stress a close integration of theoretical learning with clinical practice and interdisciplinary integration of knowledge [4, 5]. For example, CBE curricula often incorporate rich clinical experiences that allow residents to train in real healthcare settings while also encouraging participation in research projects to cultivate scientific thinking and innovation. In addition, such programs emphasize interdisciplinary collaboration; for instance, radiology residents working with surgery, internal medicine, and other departments in multidisciplinary team (MDT) activities to improve residents’ interdisciplinary communication and teamwork skills [6].

In China, medical schools and teaching hospitals have recognized the limitations of the traditional training model and have begun to explore new educational concepts and methods. Some institutions have introduced case-based learning, simulation training, and other active-learning strategies into radiology curricula. By analyzing real clinical cases and practicing with simulated procedures, these programs aim to enhance residents’ clinical reasoning and practical skills [3]. Other hospitals have partnered with universities to establish standardized radiology residency training programs and formulate comprehensive training plans that cover theoretical studies, clinical practice, research training, with the aim of cultivating radiology professionals with well-rounded skills [7, 8].

Despite various CBE-based innovations and reform efforts worldwide [9], radiology residency training continues to face persistent challenges, including high resource demands, difficulties in standardization, complex competency assessment, and barriers to adaptation for both faculty and trainees. A practical, competency-driven, and systematic training model has yet to be established. To address these gaps, we developed and evaluated a novel competency-based, disease-focused modular training model. This model integrates a cross-disciplinary learning platform, team-based clinical training, and image post-processing skills development, aiming to cultivate radiologists with strong clinical competence, research capability, and comprehensive skills aligned with evolving professional demands.

## Materials and methods

### Study design

This study was approved by the institutional review boards of West China Hospital of Sichuan University (20210807), and written informed consent was obtained from all participants prior to enrollment. We conducted a controlled study to compare the effects of a competency-based, disease-specific modular training model with those of the traditional training model on the educational outcomes of radiology residents. The study participants included radiology residents in the classes of 2021 and 2022 (assigned to the experimental group) and those from the classes of 2019 and 2020 (assigned to the control group). All included participants were entering their second year of standardized residency training. The participants were not assigned to fixed subspecialty tracks at baseline; rather, they were radiology residents who rotated through multiple subspecialty teams as part of the training program.

For subgroup analysis, residents were divided into an enrolled-graduate subgroup and a non-graduate subgroup according to whether they were concurrently enrolled in a graduate-degree program during residency training. In this study, the enrolled-graduate subgroup referred to residents who were simultaneously undertaking standardized residency training and a master’s or doctoral degree program, whereas the non-graduate subgroup referred to residents who entered residency directly without concurrent graduate-degree enrollment, most commonly after bachelor’s-level medical education.

**Fig. 1:**
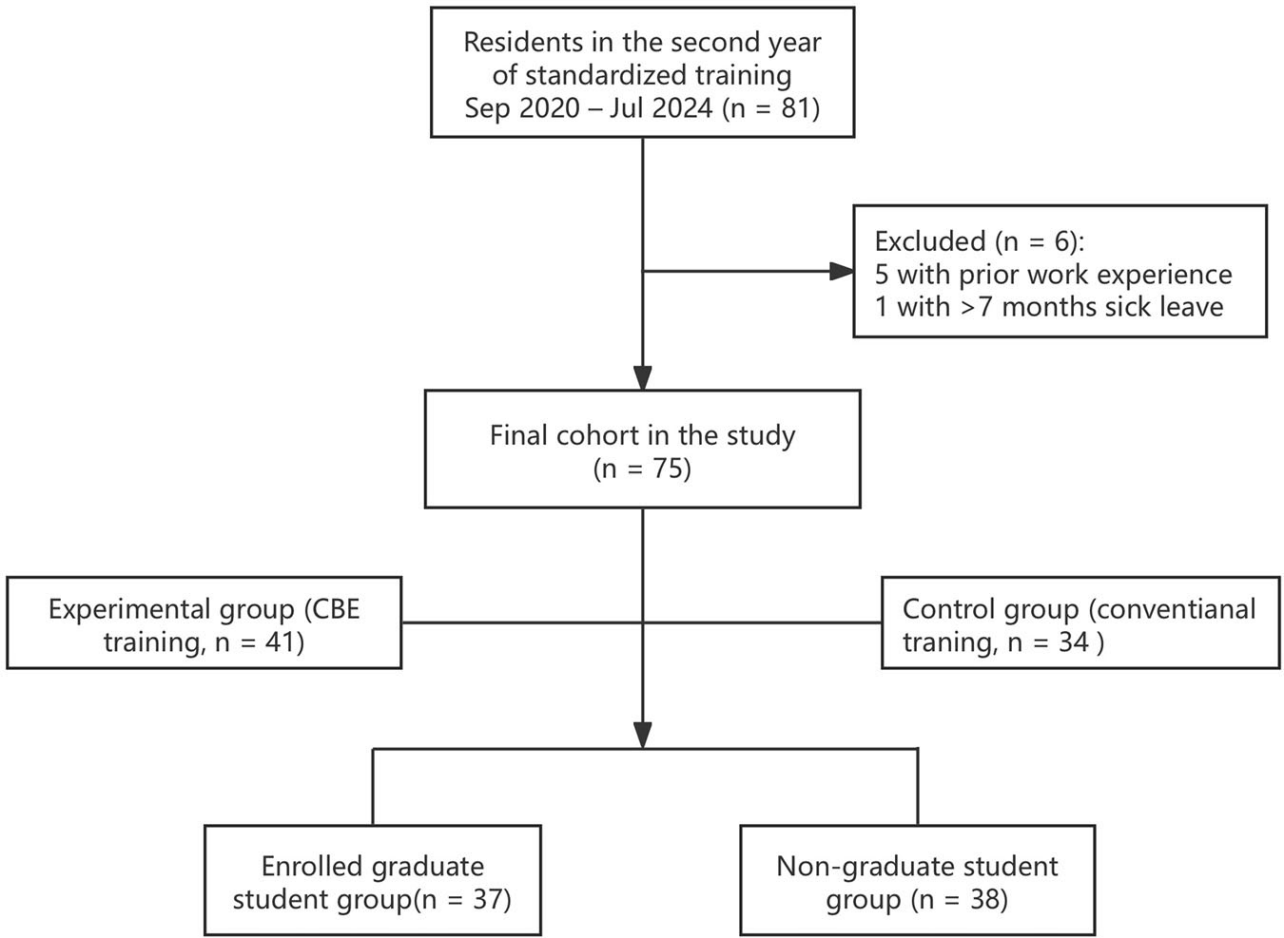
Flowchart of participant enrollment and group allocation. This figure illustrates the enrollment and exclusion process for radiology residents in the study, as well as the allocation into experimental (CBE training) and control (conventional training) groups, with subgroup analysis based on graduate status

The flowchart of participant inclusion in the study is shown in Fig. 1. This study was approved by the institutional review board (IRB), with approval granted in June 2021.

### Training intervention based on the CBE model

**Fig. 2:**
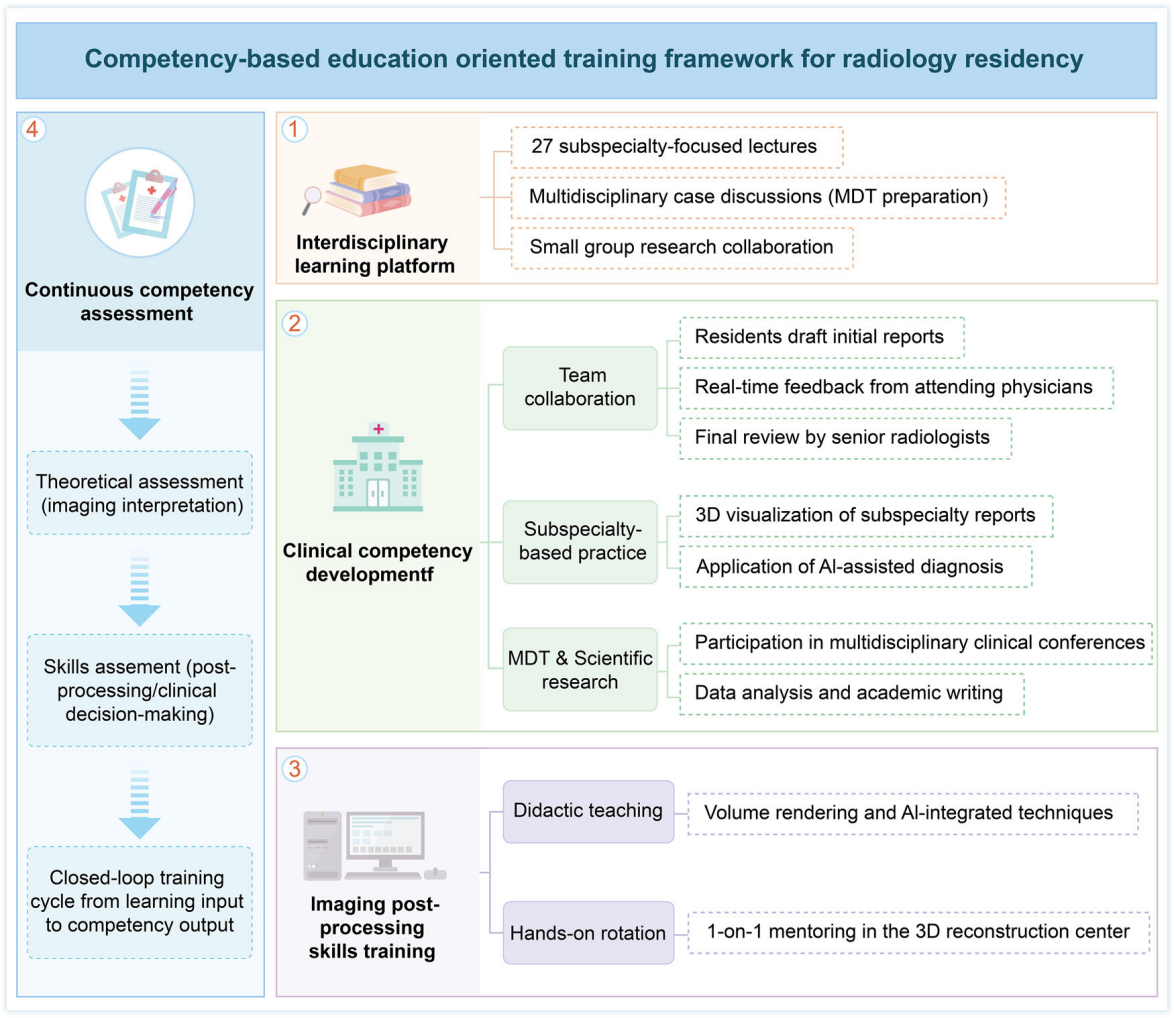
Framework of the CBE oriented modular training model. This diagram illustrates the CBE-based training model developed for radiology residents. The model consists of three interconnected modules: (1) an interdisciplinary learning platform, (2) clinical competency development through subspecialty-based rotations and teamwork, and (3) imaging post-processing skills training. These modules are integrated within a continuous competency assessment system designed to create a closed-loop training cycle from learning input to competency output

The experimental group received a new training program that integrated technology, diagnostics, radiology, and clinical practice components within a CBE-oriented model. This program consisted of three major elements: (1) a cross-disciplinary learning platform, (2) clinical training in specialized teams, and (3) imaging post-processing skills training. The framework of the CBE-oriented modular training model is shown in Fig. 2.

#### Cross-disciplinary learning platform

The cross-disciplinary learning platform delivered teaching in a variety of formats, including specialty lectures, case discussions, and group learning, which were led by senior radiologists heading each of the 27 disease-focused subspecialty teams in the radiology department. Each teaching session lasted one hour. This curriculum not only covered core radiology knowledge but also integrated content from clinical medicine, basic science, biomedical engineering, and other fields, enabling residents to develop a holistic understanding of diseases and the latest technological advances.

For example, in a hepatobiliary imaging module, residents first reviewed the clinical presentation and pathophysiology of portal hypertension with clinicians and radiologists and then learned the biomedical engineering principles underlying computed tomography (CT) angiographic reconstruction, three-dimensional vascular segmentation, and image post-processing. They subsequently applied these techniques at the workstation to reconstruct portal venous anatomy, evaluate lesion-vessel relationships, and discuss how processed images could support diagnostic interpretation and treatment planning. This case-based design allowed biomedical engineering concepts to be taught in a clinically relevant and application-oriented manner.

*Interdisciplinary learning environment*: The platform provided an environment for multidisciplinary learning, covering 27 disease-oriented modules spanning all major body systems. It integrated fundamental radiological knowledge and skills with relevant concepts from clinical medicine, basic sciences, and biomedical engineering, with the goal of cultivating residents’ broad medical knowledge and systems thinking.

*Diverse teaching methods and research cultivation*: A wide range of teaching activities, including lectures, case discussions, and small-group learning, were employed to stimulate residents’ scientific thinking and creativity. Instructors guided trainees in formulating questions, analyzing problems, and seeking solutions, thereby developing their research skills and innovative thinking and laying a solid foundation for future scholarly work.

#### Clinical team-based training

In the experimental model, we implemented a disease-specific, MDT-based approach to clinical training. Under this approach, each resident rotated through different radiology subspecialty teams, such as neuroradiology and musculoskeletal imaging, thoracic, abdominal, and emergency imaging, under the guidance of a dedicated mentor, while a continuous imaging service management system was in maintained. This design strengthened residents’ clinical skills and fostered teamwork. The team-based training involved several key features:

*Teamwork and immediate feedback*: All members of the team, including residents, junior attending radiologists who performed preliminary image reviews, and mentors (senior radiologists), worked together in the same workspace. This co-location facilitated real-time communication and feedback between trainees and supervisors, allowing errors to be promptly corrected and enhancing learning efficiency.

*Specialized practice and MDT participation*: Residents actively took part in the team’s disease-specific clinical activities, which included interpreting images, drafting specialized imaging reports, and participating in MDT discussions with other departments. Preparing subspecialty imaging reports required a higher level of expertise, often involving advanced three-dimensional image visualization and the application of AI tools in image analysis.

*Research and teaching skill development*: Residents were engaged in academic activities such as literature review, data collection, analysis, and manuscript writing. They also contributed to the team’s educational tasks, for example, by preparing cases for weekly difficult-case discussions, writing case study reports, and delivering short teaching sessions to peers. These activities helped cultivate residents’ research awareness and improve their teaching, presentation, and communication skills.

#### Imaging post-processing skills training

Training in imaging post-processing was another critical component of the experimental curriculum, comprising both didactic learning and hands-on practice. The aim was to increase residents’ proficiency in advanced image processing techniques and familiarize them with modern imaging technologies, including AI applications in imaging.

*Theoretical instruction*: Residents first attended a dedicated 2-h lecture covering the principles of various image post-processing techniques. The content included volume rendering, maximum-intensity projection, minimum-intensity projection, multiplanar and curved planar reconstructions, CT and magnetic resonance imaging (MRI) angiographic reconstructions, CT perfusion imaging, and MRI functional imaging post-processing. The didactic session ensured that residents understood how each technique was applied in clinical practice, thereby laying a strong theoretical foundation for subsequent hands-on training.

*Hands-on practice*: After the lectures, residents underwent a one-month rotation in a three-dimensional (3D) image post-processing laboratory. Using a small-group, apprenticeship-style format, experienced instructors provided one-on-one guidance at the workstation. Residents started with basic operations and gradually learned to perform all the aforementioned post-processing techniques, effectively translating their theoretical knowledge into practical skills. Through this rotation, residents became proficient in using imaging software and were able to independently complete common post-processing tasks such as 3D reconstructions, image fusion, and functional imaging analyses.

### Training intervention based on the traditional model

Residents in the control group were trained under the standard radiology residency curriculum traditionally used in China, which combines didactic coursework with routine clinical rotations. The lecture-based coursework covers the imaging manifestations of common diseases in all organ systems, with an emphasis on delivering foundational knowledge. Clinical training follows a rotation system in which residents rotate through subspecialty divisions (e.g., neuroradiology and musculoskeletal imaging, cardiovascular and thoracic imaging, abdominal imaging, and emergency radiology), typically spending approximately three months in each subspecialty. During these rotations, residents serve as primary report drafters in the assigned section; their work location, case assignments, and supervising attendings follow the usual departmental scheduling without assignment to a fixed mentorship team.

### Outcomes assessment

Both the experimental and control groups were trained within the same radiology department, and the teaching faculty were drawn from the same pool of attending and senior radiologists. Therefore, the principal difference between the two groups lay in the training organization and instructional model rather than the faculty background.

The primary outcomes were the final theoretical and practical test scores, both assessed immediately after completion of the training program. Each examination was scored on a 100-point scale. The theoretical examination assessed residents’ mastery of core medical imaging knowledge, whereas the practical examination evaluated competence in image interpretation, image post-processing, and clinical diagnostic decision-making. In addition, both groups were evaluated using the same examinations and the same scoring criteria.

### Statistical analysis

All data were analyzed using SPSS version 26.0. The Kolmogorov‒Smirnov test was used to examine the normality of the score distributions. Continuous variables with a normal distribution were expressed as the mean ± standard deviation (SD), and those without a normal distribution are expressed as the median (IQR). An independent-samples *t*-test or a Mann‒Whitney *U*-test was used to compare the theoretical and practical scores between the experimental and control groups, as appropriate. A *p*-value < 0.05 was considered statistically significant.

## Results

### Study participants

As shown in Fig. 1, from September 2020 to July 2024, a total of 81 radiology residents entering their second year of standardized residency training at our institution were screened. Five of these residents were excluded because they had prior work experience, and one resident was excluded because of taking sick leave for longer than 7 months. Ultimately, 75 residents were enrolled in the study (22 males and 53 females; mean age 24 ± 2.18 years, range 22–33 years). Among these, 41 residents underwent the CBE-based modular training (experimental group), and 34 received traditional training (control group). For subgroup analysis, 37 residents were classified into the enrolled-graduate subgroup and 38 into the non-graduate subgroup according to educational background. The baseline characteristics of the experimental and control groups are presented in Table 1.

**Table 1:** Baseline characteristics of the residents

	Experimental group	Control group	*p*
Gender			< 0.001
Male	10	12	
Female	31	22	
Age (years)	24.71 ± 2.247	24.62 ± 2.035	0.14
Degree status			< 0.001
Non-graduate resident	24	14	
Enrolled graduate student	17	20	

Data are presented as the number of residents or mean ± SD. *p*-values were calculated using the chi-square test for categorical variables and the independent *t*-test for continuous variables

### Exam performance

The examination outcomes of the experimental and control groups are summarized in Table 2. Overall, the two groups did not differ significantly in overall examination performance. The mean theoretical knowledge examination score in the experimental group was 83.26 ± 6.99, whereas that in the control group was 81.76 ± 7.54 (*p* = 0.39). The mean practical skills examination score was 86.79 ± 8.90 in the experimental group and 84.91 ± 10.71 in the control group (*p* = 0.41).

**Table 2:** Comparison of assessment scores between experimental and control groups

Assessment group	Experimental group	Control group	*p*
Overall performance			
Theoretical score	83.26 ± 6.99	81.76 ± 7.54	0.39
Practical score	86.79 ± 8.9	84.91 ± 10.71	0.409
Non-graduate residents			
Theoretical score	81.38 ± 6.76	75.57 ± 6.89	0.0179
Practical score	81.23 ± 7.33	75.29 ± 8.42	0.028
Graduate student residents			
Theoretical score	86.11 ± 5.46	86.09 ± 4.29	0.992
Practical score	95.5 (94.0–97.0)	93.0 (88.0–95.5)	0.092

Data are shown as mean ± SD or median (IQR). *p*-values were calculated using an independent *t*-test or Mann–Whitney *U*-test where appropriate

**Fig. 3:**
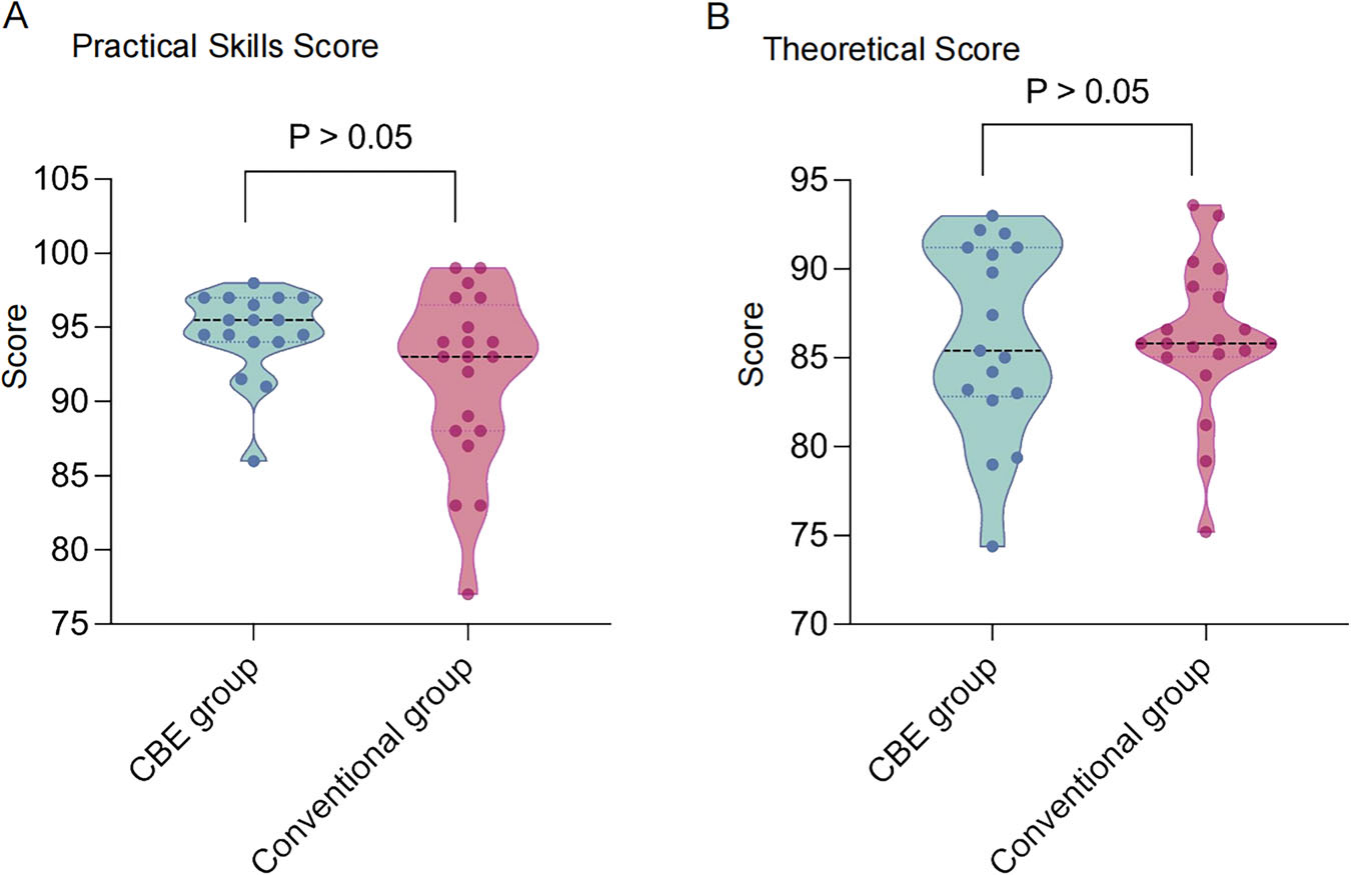
Comparison of practical and theoretical scores between CBE and conventional groups in the enrolled-graduate residents. Violin plots show the distribution of **A** practical and **B** theoretical examination scores among enrolled-graduate residents. No statistically significant differences were found between the CBE and conventional training groups (*p* > 0.05)

**Fig. 4:**
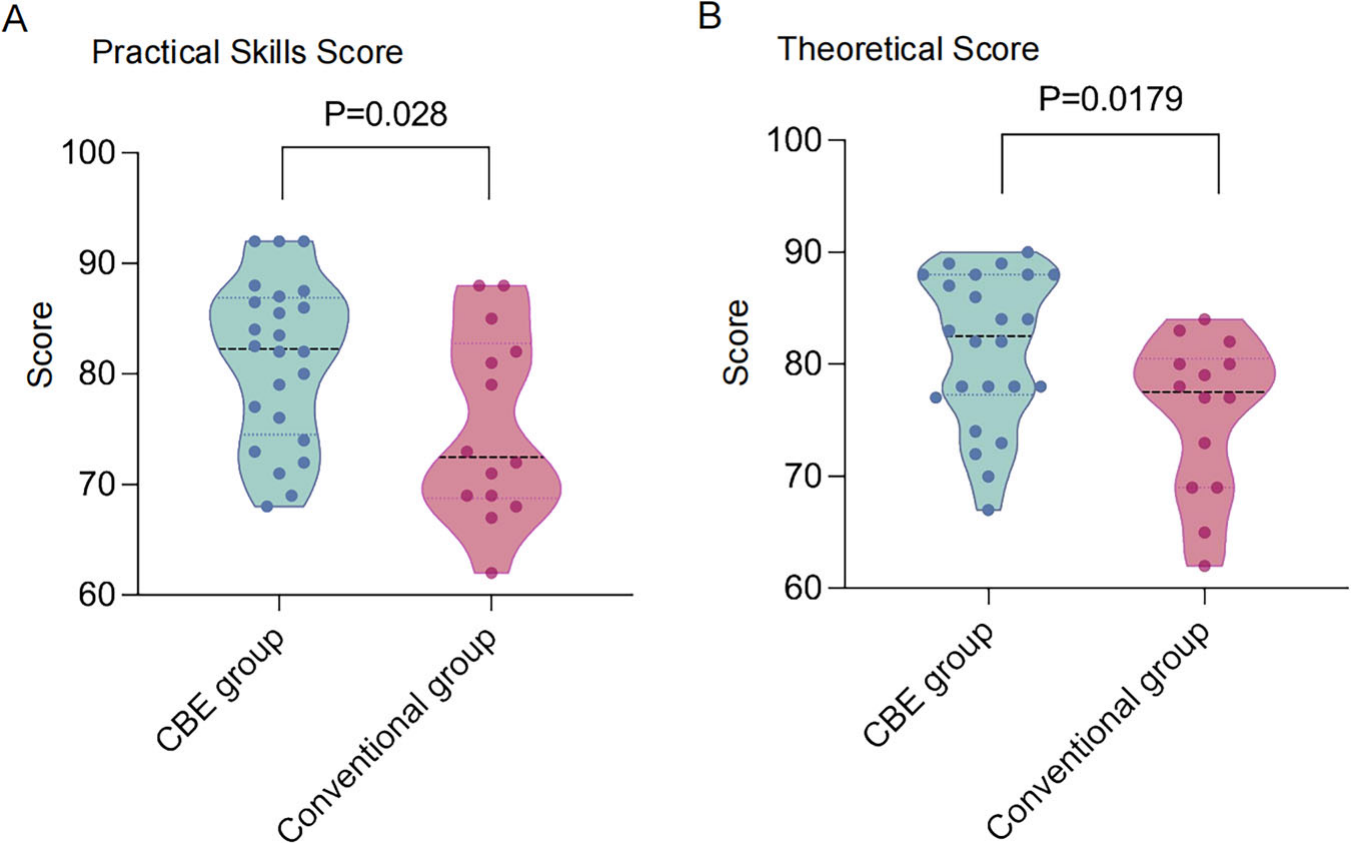
Comparison of practical and theoretical scores between CBE and conventional groups in non-graduate residents. Violin plots display **A** practical and **B** theoretical test scores for non-graduate residents. The CBE group demonstrated significantly higher performance than the conventional group in both practical (*p* = 0.028) and theoretical (*p* = 0.0179) assessments

In subgroup analyses, residents in the enrolled-graduate subgroup showed no significant differences between the experimental and control groups in either the theoretical examination (experimental 86.11 ± 5.46 vs control 86.09 ± 4.29, *p* = 0.992) or the practical examination (median 95.5 [94.0–97.0] vs 93.0 [88.0–95.5], *p* = 0.092) (Fig. 3). In contrast, among residents in the non-graduate subgroup, those in the experimental group achieved significantly higher scores than those in the control group on both assessments: for the theoretical examination, the scores were 81.38 ± 6.76 vs 75.57 ± 6.89 (*p* = 0.018); for the s practical examination, the scores were 81.23 ± 7.33 vs 75.29 ± 8.42 (*p* = 0.028) (Fig. 4).

## Discussion

In this study, we developed a disease-oriented, competency-based training model for radiology residency that integrates interdisciplinary coursework, team-based clinical training, and imaging post-processing instruction. Although overall examination scores did not differ significantly between the competency-based and traditional training programs, subgroup analysis indicated that the new model was particularly beneficial for residents in the non-graduate subgroup, namely those who entered residency without concurrent graduate-degree enrollment, as they achieved significantly higher theoretical and practical scores than those in the traditional training program. This finding suggests that the CBE-based modular curriculum may be especially valuable for trainees with relatively limited concurrent academic training during residency. Although the Chinese system of undergraduate medical education and residency training differs from that of many other countries, the core elements of this model, including structured competency-based progression, interdisciplinary integration, and clinically oriented modular teaching, may still offer useful insights for residency training in other institutional settings.

The greater benefit observed in the non-graduate subgroup may be explained by differences in prior training exposure between the two educational subgroups. In the Chinese training context, residents in the non-graduate subgroup generally entered standardized residency training directly without concurrent graduate-degree enrollment, whereas residents in the enrolled-graduate subgroup simultaneously undertook residency training and a master’s or doctoral degree program [9]. Compared with residents in the enrolled-graduate subgroup, those in the non-graduate subgroup may have had fewer opportunities for structured research participation, interdisciplinary learning, and the development of higher-level clinical reasoning. Therefore, the CBE-oriented modular curriculum used in this study, which integrates cross-disciplinary teaching, supervised team-based practice with immediate feedback, research engagement, and image post-processing training, may have provided greater incremental benefit for this subgroup. By contrast, residents in the enrolled-graduate subgroup may already have acquired some of these competencies through their graduate training and may also have had stronger self-directed learning abilities, leaving less room for measurable improvement and reducing the additional challenge provided by the curriculum [10].

Our competency-oriented, disease-specific curriculum was designed to train radiologists who are well-equipped for contemporary developments in the field and current clinical demands. However, in some hospitals in China, including large institutions, radiology residents often receive only general radiology training without subspecialty education, as there is no formal subspecialization in the field. Notably, through carefully structured multidisciplinary coursework, tightly integrated theoretical and practical training, and the parallel emphasis on specialty clinical practice and research, the program aimed to cultivate residents with a solid foundation of knowledge, strong clinical skills, and robust research capability. The model emphasizes several key elements: (1) a close integration of theory and practice, to ensure that residents not only acquire essential knowledge but can also apply it effectively in real clinical scenarios; (2) interdisciplinary learning, achieved by incorporating clinical medicine, basic science, biomedical engineering and other domains to broaden residents’ perspectives and enhance their comprehensive analytical abilities; (3) development of research skills, by encouraging residents to engage in research projects and scholarly discussions to foster scientific thinking and innovation, thereby laying the groundwork for future academic and clinical research; (4) intensive clinical skills training, including simulated clinical environments and dedicated image post-processing training, to improve residents’ procedural competencies and imaging analysis skills; and (5) a competency-driven approach to education reform, which integrates the above components into a unified system. The three modules, the cross-disciplinary platform, team-based clinical training, and post-processing training, are interlinked and mutually reinforcing. Our results indicate that this integrated model can enhance residents’ overall competencies in a comprehensive manner, helping them to independently and efficiently perform as radiologists upon graduation. Compared with the traditional approach, this model demonstrated several notable advantages, which are outlined below.

### Significance of the cross-disciplinary learning platform

The establishment of a cross-disciplinary platform broke down the conventional barriers between specialties and provided residents with a broad, integrative learning environment. Through this platform, radiology residents not only acquired radiology-specific knowledge and skills but also gained exposure to relevant knowledge in clinical medicine, fundamental biomedical science, and related disciplines. This comprehensive approach enabled residents to develop a more complete understanding of the imaging manifestations, clinical features, pathophysiology, and treatment advances of various diseases. Such interdisciplinary learning helped residents build a more systematic medical knowledge framework, improving their clinical reasoning and holistic problem-solving abilities. Moreover, the cross-disciplinary platform stimulated residents’ research thinking and innovative ideas by encouraging them to pose new research questions and pursue multidisciplinary research, thereby contributing to the advancement of radiology and related fields.

### Innovations in team-based clinical training

Traditional radiology residency training in China largely relies on rotational assignments, in which residents spend fixed periods of time rotating through different radiology subspecialties (e.g., neuroradiology, chest and abdominal imaging, and interventional radiology) to gain exposure to a range of imaging procedures and diagnoses [8]. This rotation-based model helps trainees acquire basic knowledge and skills across subspecialties, but it often lacks structured clinical mentorship and timely feedback; residents may face difficulties and challenges in clinical decision-making under this system. In contrast, many residency programs abroad place greater emphasis on developing residents’ clinical proficiency and research capacity, for example, through involvement in clinical research projects and participation in multidisciplinary care teams to sharpen clinical reasoning and research skills [6]. Furthermore, training programs in other countries widely employ advanced educational tools such as simulation training and virtual reality to offer more realistic and diverse clinical learning experiences [11]. Drawing on a wide range of practices, and addressing the shortcomings of the conventional approach, our model introduced a dedicated team-based clinical training system. In this system, residents’ clinical work was tightly integrated with education under the guidance of an appointed team leader who oversaw clinical practice, teaching, and administrative supervision. This ensured that residents received consistent, timely guidance during their rotations. The model also stressed collaboration and MDT work: by actively participating in specialized reporting tasks and MDT discussions, residents engaged with clinicians, nurses, and other healthcare professionals to deliver high-quality patient care. This not only improved the residents’ clinical skills and confidence but also cultivated their team-oriented mindset and interdisciplinary communication skills.

### Importance of post-processing training

Training in image post-processing skills is an indispensable component of modern radiology education. As imaging technology advances, post-processing techniques are playing an increasingly important role in diagnostic and therapeutic processes. Through systematic post-processing training, residents in our program became proficient in a range of advanced imaging techniques, such as 3D reconstructions, image fusion, and functional imaging analyses, which greatly increased the efficiency and depth of their image interpretation [12]. Mastery of these techniques can improve diagnostic accuracy and reliability and provide additional valuable information to aid in clinical decision-making [12]. Additionally, many post-processing methods are closely intertwined with AI technologies in imaging [13]. By learning and practicing post-processing, residents also gain insight into the current applications and future trends of AI in radiology, laying a foundation for engagement in related research and innovation. In summary, incorporating robust image post-processing training is highly beneficial for enhancing residents’ professional skill set and technical competence.

Despite its benefits, this study has certain limitations. The sample size was relatively small, which may limit the generalizability of our findings. As an initial exploratory study, our work provides preliminary evidence; in future research, we plan to expand the sample size and further evaluate the long-term educational outcomes and impacts of this training model. Another limitation is that the educational outcomes were assessed mainly by theoretical and practical examination scores. Although these measures reflect knowledge and technical skills, they may not fully capture broader competencies such as communication, teamwork, and comprehensive clinical decision-making. No formal qualitative assessments, such as structured faculty feedback or 360-degree evaluations, were included. In addition, this training model may place relatively high demands on faculty expertise, cross-disciplinary coordination, and access to advanced imaging post-processing resources, factors that should be considered when adapting the approach in other institutions.

## Conclusions

This study supports the effectiveness of an innovative competency-based, disease-focused modular curriculum for radiology residents. The program yielded notable improvements in residents’ knowledge and skills, particularly for those in the non-graduate subgroup. This model provides a practical reference point for reforming radiology residency education and has the potential to inform future training paradigms, better aligning with the rapid advancements in imaging technology and evolving clinical needs. Through the CBE-oriented approach, residents demonstrated improved knowledge and technical skills, and the curriculum may have contributed to broader development in clinical thinking, interdisciplinary collaboration, and research awareness, helping them better adapt to the evolving demands of radiology practice. In the future, with a larger sample and ongoing refinement of the curriculum, this competency-based modular approach may offer valuable insights for broader reform of radiology residency training, especially in aligning educational models with evolving clinical demands. Future longitudinal studies are needed to determine whether these gains, particularly in AI-related applications and advanced image post-processing, are retained over time and translated into routine clinical practice.

## Abbreviations

**AI**: Artificial intelligence
**CBE**: Competency-based education
**CT**: Computed tomography
**IQR**: Interquartile range
**MDT**: Multidisciplinary team
**MRI**: Magnetic resonance imaging
**SD**: Standard deviation

## Declarations

### Methodology

- Prospective
- Observational
- Single institution

## References

[1] Zhang J, Han X, Yang Z et al (2021) Radiology residency training in China: results from the first retrospective nationwide survey. Insights Imaging 12:25. 10.1186/s13244-021-00970-2 33595737 10.1186/s13244-021-00970-2 PMC7889775

[2] Li R, Yi KM, Xiong KL (2017) Objectives and significance of standardized residency training for radiology residents. Chinese J Med Edu Res 16:610–613. 10.3760/cma.j.issn.2095-1485.2017.06.017

[3] Lu T, Peng XG, Zhao Z, Xie B, Teng GJ, Ju SH (2018) Exploration and practice of constructing a competency-based curriculum system for standardized residency training in radiology. Chin J Grad Med Edu 2:438–441

[4] Slanetz PJ, Cummings R, Gomez E, McKinney JM (2025) The debate over competency-based education in radiology—promises and perils. J Am Coll Radiol 22:691–693. 10.1016/j.jacr.2024.11.026 39612973 10.1016/j.jacr.2024.11.026

[5] Touchie C, ten Cate O (2016) The promise, perils, problems and progress of competency-based medical education. Med Educ 50:93–100. 10.1111/medu.12839 26695469 10.1111/medu.12839

[6] Bentley H, Vo CDQ, Zaki-Metias K, Nikpanah M (2023) Competency-based medical education in radiology graduate medical education: overview and future perspectives. Radiographics 43:e220197. 10.1148/rg.220197 37053101 10.1148/rg.220197

[7] Liu ZH, Yan F, Xian JF et al (2018) Evaluation of the application of an integrated PBL and LBL teaching model in medical imaging. Chin J CT MRI 16:147–149. 10.3969/j.issn.1672-5131.2018.01.045

[8] Huang RB, Ma HJ, Xu WX, Huang XX, Liu Y (2018) Exploration and experiences of standardized residency training in radiology department. Chin J Graduate Med Edu 2:204–207

[9] Lio J, Ye Y, Dong H, Reddy S, McConville J, Sherer R (2018) Standardized residency training in China: the new internal medicine curriculum. Perspect Med Educ 7:50–53. 10.1007/s40037-017-0378-5 29098637 10.1007/s40037-017-0378-5 PMC5807259

[10] Delavari S, Barzkar F, Rikers RMJP et al (2024) Teaching and learning clinical reasoning skill in undergraduate medical students: a scoping review. PLoS One 19:e0309606. 10.1371/journal.pone.0309606 39413083 10.1371/journal.pone.0309606 PMC11482728

[11] Yang J, Jomaa D, Islam O, Mussari B, Laverty C, Kwan BYM (2021) Competency-based medical education in radiology: a survey of medical student perceptions. Can Assoc Radiol J 72:352–358. 10.1177/0846537119893663 32103685 10.1177/0846537119893663

[12] Gondim Teixeira PA, Cendre R, Hossu G et al (2017) Radiology resident MR and CT image analysis skill assessment using an interactive volumetric simulation tool—the RadioLOG project. Eur Radiol 27:878–887. 10.1007/s00330-016-4384-5 27165134 10.1007/s00330-016-4384-5

[13] Salvi M, Acharya UR, Molinari F, Meiburger KM (2021) The impact of pre- and post-image processing techniques on deep learning frameworks: a comprehensive review for digital pathology image analysis. Comput Biol Med 128:104129. 10.1016/j.compbiomed.2020.104129 33254082 10.1016/j.compbiomed.2020.104129

